# Epidemiology of Leptospirosis in Costa Rica 2011–2015

**DOI:** 10.1007/s40475-017-0102-x

**Published:** 2017-03-14

**Authors:** Marcelo Pérez Carvajal, Kaila A. Fagerstrom

**Affiliations:** 1 Consejería y Atención Médica, Residencia las Palmeras, casa #1, Daniel Flores, San José, Pérez Zeledón Costa Rica; 2 National School of Tropical Medicine, Baylor College of Medicine, 1102 Bates St. Feigin Bldg, Suite 0550, Houston, TX 77030 USA

**Keywords:** Leptospirosis, Costa Rica, Epidemiology, Control programs, Surveillance, INCIENSA (Instituto Costarricense de Investigación y Enseñanza en Nutrición y Salud)

## Abstract

**Purpose of Review:**

Leptospirosis is a global spirochete causing chronic renal disease that is increasing in Costa Rica. This paper identifies the prevalence and risk factors of leptospirosis in Costa Rica between the years of 2011–2015.

**Recent Findings:**

Clinical cases of leptospirosis in Costa Rica demonstrated various symptoms: from asymptomatic diseases to severe cases of kidney and liver failure. A variety of diagnostic methods with varying specificities and sensitivities were employed. In Costa Rica, prevention methods such as protective clothing, decreased contact with animals, and prophylaxis of close contacts continue to be the most important factors in reducing transmission of leptospirosis.

**Summary:**

In Costa Rica, the following populations should be aware of their increased risk: those living in the province of San José, Puntarenas, or Alajuela; being a male; being of productive years; and exposure to specific environmental factors.

## Introduction

Leptospirosis is a globally distributed zoonotic disease that may cause severe complications or death [1, 2]. This disease is often endemic and asymptomatic in tropical areas such as Costa Rica. Epidemics may occur, especially with sudden rainfall or flooding, as the increased amount of water enables the spread of the spirochete [3].

Several host reservoirs exist, with the brown rat (*Rattus norvegicus*) being the most common for human infection [4]. While leptospirosis is typically presented as a nonspecific acute febrile disease, it can leave a devastating impact on patients. A typical clinical presentation consists of fever, myalgia, and headache. This can progress in severe patients into a cytokine storm, multisystem organ failure, oliguric renal failure, and hemorrhagic complications such as severe pulmonary hemorrhagic syndrome, which can have a death rate of >50% [5].

While leptospirosis has a global distribution, its economic burden of disease is noteworthy, especially in tropical countries like Costa Rica. Research reveals that highest-burden estimates occur in tropical countries with fewer resources. In these tropical regions, leptospirosis is noted as being “underappreciated” as an economic burden as more focus may be on other febrile diseases such as dengue [5].

With the appearance of dengue in the 1990s in Costa Rica, detection of leptospirosis cases increased as a default from the overall improved diagnostics of febrile cases. Accordingly, three government institutions came together to target surveillance of leptospirosis in Costa Rica: Costa Rican Department of Social Security (CCSS), the Costa Rican Ministry of Health’s (INCIENSA) leptospirosis laboratory, and the Ministry of Agriculture and Ranching’s laboratory of animal health. In Costa Rica, screening occurs for leptospirosis in patients with (1) a sudden fever of 38 °C/100.4 °F, (2) less than 7 days of development, and (3) in which no other infectious agent is suspected [6]. All private and public laboratories are required to send all samples of suspect leptospirosis cases to INCIENSA, the national center of reference of leptospirosis for diagnostics [6].

Since reporting of the disease to INCIENSA is mandatory, a publicly accessible database was available for our analysis of the years 2011–2015. It is important to note that before 2013, microscopic agglutination test (MAT) was the only diagnostic test used to detect positive cases despite several other diagnostic methods that exist with varying specificities and sensibilities [7•]. After 2013, polymerase chain reaction (PCR) was implemented in addition to MAT, as PCR has greater success in acute phase detection of the disease. In this article, we analyzed the epidemiologic variables and risk factors of confirmed leptospirosis cases obtained by INCIENSA in the last 5 years. Specifically, we evaluated the seasonal quarter, years, provinces with the greater number of positive cases and suspect cases, skill of the provincial diagnostic capacities, gender, age, and other risk factors that promote differences of positive samples for leptospirosis. Each of these data points was analyzed by year, in order to describe the epidemiological profile of leptospirosis in Costa Rica.

## Suspect and Confirmed Cases of Leptospirosis by Year, Costa Rica, 2011–2015

In the last 5 years, 5056 samples of leptospirosis have been sent to INCIENSA to be processed and 536 of the suspected cases were confirmed as positive for leptospirosis, resulting in a 10.6% diagnostic success. The years with the greatest number of suspect samples are 2011, with 2003 suspect samples sent to INCIENSA, and 2014, with 1327 suspect cases. In 2012 and 2015, the suspect cases were much less, as seen in Table 1 [8, 9, 12–14].

**Table 1:** Suspect positive and negative cases of leptospirosis and their diagnostic success, Costa Rica, 2011–2015

	Positive cases	Negative cases	Total cases	Diagnostic success (%)
2011	228	1776	2003	11.38
2012	47	446	493	9.53
2013	71	809	881	8.05
2014	137	1190	1327	10.32
2015	53	299	352	15.0

Source: Self-evaluated using INCIENSA data

In 2011, for every 100 suspect samples, 11 confirmed as positive for leptospirosis resulting in an 11.3% diagnostic success. In 2012 and 2013, however, the confirmed diagnosis was less than 10%, and in 2015, despite having the fewest number of suspect samples, the greatest diagnostic success percentage occurred with 15.0% of suspected cases resulting in a positive diagnosis as seen in Table 1 [8, 9, 12–14]. Overall, the year with the greatest number of suspect and positive cases was 2011.

## Suspect and Confirmed Cases of Leptospirosis by Season, Costa Rica, 2011–2015

The season with the most suspect and confirmed cases for leptospirosis in Costa Rica changed yearly between 2011 and 2015, suggesting a strong environmental influence on leptospirosis exposure and physician suspicion. The leptospirosis spirochete has a corkscrew-like tail enabling it to maneuver through water easily. Thus, in seasons with extensive rainfall, overflowing rivers and lakes, and flooding, the likelihood that the spirochete has human and animal exposure is increased. We see this evidenced in the yearly cases according to seasonal quarter.

In 2011, 2003 suspect cases of leptospirosis occurred throughout Costa Rica. The greatest number of suspect cases occurred during the last quarter of the year, October through December, with a total of 614 (40%) of the cases. During the remaining quarters of the year, the distribution of the suspect cases was homogenous.

In 2012, INCIENSA reported 493 suspect cases with 47 confirmed positive cases of leptospirosis for the year, all of which occurred during the January through March quarter. The remaining quarters had no suspect cases reported to INCIENSA [9].

In 2013, 881 suspect case samples were sent to INCIENSA for the second and third quarters of the year, April through September. In the second quarter, between April and June, 386 suspect cases were reported with 36 positive results. In the third quarter, between the months of July through September, 423 suspect cases were reported with 35 positive cases. Similar to 2012, no data is found on the official INCIENSA site for the remaining quarters of the year. Of these 881 cases in 2013, 71 (8%) were positive.

In 2014, INCIENSA processed 1327 samples. Throughout July to September, the greatest number of suspect cases was reported for that year, 397 total. This data, however, does not signify that in these months the greatest number of positive cases was obtained; in the first quarter, 307 leptospirosis suspect samples were reported, with 45 positive cases, making the first quarter of 2014 the quarter with the greatest number of positive cases [13].

In 2015, suspect cases were only reported during the first quarter of the year with a total of 352 samples processed by INCIENSA, of which 53 were positive cases [14].

**Fig. 1:**
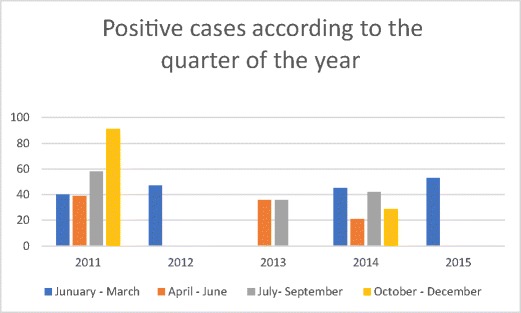
Positive cases of leptospirosis according to quarter of the year, Costa Rica, 2011–2015. Source: Self-evaluated using INCIENSA data

Overall, between 2011 and 2015, the season with the most positive samples has varied as seen in Fig. 1. In 2011, the last quarter of the year had the greatest number. In 2012, positive cases were only reported in the first quarter of the year. In 2013, only the second and third quarters of the year reported cases. In 2014, the first quarter had the most positive cases and the third quarter had the most suspect cases. In 2015, again only the first quarter reported any suspect or confirmed cases. With this data, we can conclude that the different seasons over the last 5 years, with respect to the number of positive cases/quarter of the year, are influenced from environmental factors that facilitate the contact of the spirochete with humans [12].

## Suspect and Confirmed Cases of Leptospirosis by Province, Costa Rica, 2011–2015

As the seasons of the year vary in reporting leptospirosis cases, the regions of Costa Rica follow suit. Several provinces stand out as leading leptospirosis regions, while others report few cases across the years.

The confirmed positive cases by MAT demonstrate that San José is the province with the most reported cases as well as positive cases in 2011, with 19.12% diagnostic success for the region and year. The province of Guanacaste reported 134 suspect cases, of which 23 were positive, for a 17.16% of the total number of samples for its province, placing it in second place with the highest leptospirosis outcomes. In the province of Limón, the number of suspect leptospirosis samples sent to INCIENSA is noteworthy, 236 samples, of which only 10 were positive, representing 4.23% of the sent samples for the province [8]. The province of Heredia, on the other hand, is the province that reports less suspect cases and less confirmed samples by MAT during 2011. It is not clear if Heredia’s low positive samples were due to less suspect cases from the diagnostic in the province or because it has a lesser number of risk factors for the population.

The province with the most suspect and positive cases in 2012 was again San José, with a total of 25 confirmed cases by MAT between January and March. Within this province, it is interesting to note that the region with the most suspect cases is Brunca and more specifically the town of Pérez Zeledón [9]. The province of Heredia is a province with known emphasis on screening for leptospirosis. Nonetheless, of the total number of suspect cases for Heredia in 2012, none were found to be positive. Similarly, the province of Limón also stresses leptospirosis testing. Of its 62 suspect cases for leptospirosis, only one was positive. In Limón, it is interesting to note that dengue testing is a frequent diagnostic in emergency services. Clinically, dengue presents signs and symptoms similar to the febrile symptoms of leptospirosis. Based on the INCIENSA criteria to distinguish for leptospirosis, it is very probable that many patients in Limón with suspected dengue submitted leptospirosis samples as well, signifying that in this province, there were many reported suspect cases and very few positive leptospirosis cases [9, 10••].

In 2013, the province of Puntarenas dominates first place with 25 positive cases followed by San José with 14. Heredia maintains with only one positive case reported in 2013 [12].

2014 is similar to the previous years in which San José and Puntarenas are first in the greatest number of positive cases, 52 and 22, respectively. Heredia, again, is the province with the least number of positive cases (*n* = 6), and Guanacaste trails with 7 positive cases in 2014 [12].

San José is the leader in 2015 with 23 positive leptospirosis samples. Alajuela, Limón, and Puntarenas all tie for second place with 8 positive samples. As expected, Heredia reported only 1 positive sample, as well as Cartago. 2015 data was interesting, as Limón stands out from previous years, with a considerable increase of positive samples for leptospirosis. This may point toward a natural phenomenon, such as floods and overflowing rivers which are common in this province, increasing the exposure of leptospirosis to humans [14].

Overall, San José is the province which has the greater number of positive samples over the 5 years, with a total of 197 positive cases of the 536 samples, representing 37%. At the same time, Heredia and Guanacaste possessed a smaller number of positive samples with 11 (2%) and 27 (5%), respectively [8, 9, 12–14].

## Suspect and Confirmed Cases of Leptospirosis by Age and Gender, Costa Rica, 2011–2015

In 2011, the greater number of suspect and positive cases of leptospirosis occurred in males [8]. Upon looking at the analysis of seasonal quarters by age and gender, one notes interesting variations. The age of males with positive samples most notable during the January to March quarter are those within the age group of 31–40 years old with a total of 10 positive cases. Meanwhile, for females in the same quarter, the age with the greatest number of positive cases is the age group of 51–60 years old, which is consistent throughout nearly all of 2011. Nonetheless, during the months of April–September for males, the age with the greatest number of positive cases presented occurred in the age range of 11–20 years old, with a total of 13 confirmed samples using MAT in INCIENSA. The end of the year is when more positive cases were reported for males, with ages comprised between 41 and 50 years old, with a total of 20 confirmed cases by MAT compared to just two cases confirmed by MAT with females of the same time period with ages between 50 and 60 years. Overall, for 2011, we can conclude that the age groups represented seasonally and by gender varied, but the majority of cases occurred in males and during productive years [8].

Commonly in private hospitals, EBAIS, and other Costa Rican health centers, more suspect leptospirosis cases are reported in males compared to females of samples sent to INCIENSA. In fact, of the 881 reported suspect cases in 2013, 615 were in males, totaling 70% of samples processed by INCIENSA, coinciding with the risk factor that positive leptospirosis males are represented more than females [11, 12]. In regard to age, only two reported positive samples of children under the age of 10 years old were reported in 2013, the only year reporting cases for that age group. In males, the number of positive cases from age 11 to 50 years old increases as observed in previous years. In 2013, however, the number of positive samples extended to 60 years old instead of 50 [12]. In females, the data varied considerably. In the previous 2 years, the greatest number of positive cases was presented between the ages of 51 to 60 years old, while in 2013, the age of the most total cases changed to 21 to 30 years old [12].

In regard to gender in 2014, no changes occur in relation to previous years, where the number of suspect cases for leptospirosis in males tripled that of females. For both males and females in 2014, no positive cases are reported between 0 and 10 years old and positive cases begin increasing from 11 years old and decreasing at 60 years old [13].

In 2015, again, leptospirosis is suspected and confirmed more commonly in males [14]. When referring to age for 2015, we see that very few positive samples for leptospirosis exist in the extremes of life for both sexes. The number of positive samples starts to increase after age 11 and diminish after age 50 [14].

The following table, Table 2, represents the distribution of positive leptospirosis cases by age, gender, and year analyzed.

**Table 2:** Positive leptospirosis cases according to age and gender, Costa Rica, 2011–2015

Age	Gender	2011	2012	2013	2014	2015	Total
0–10	Male	7	0	2	0	0	9
	Female	1	0	0	0	0	1
11–20	Male	51	11	6	13	8	89
	Female	3	0	1	4	2	10
21–30	Male	45	8	13	21	20	107
	Female	2	0	6	3	0	11
31–40	Male	28	9	19	23	6	85
	Female	2	0	3	7	2	14
41–50	Male	33	12	5	27	9	86
	Female	3	0	1	1	0	5
51–60	Male	28	1	7	23	4	63
	Female	6	3	2	4	0	15
61–70	Male	12	2	2	6	2	24
	Female	0	0	0	2	0	2
>70	Male	2	1	3	3	0	9
	Female	0	0	0	0	0	0
Without data	Male	2	0	0	0	0	2
	Female	3	0	1	0	0	4

Source: Self-evaluated using INCIENSA data

## Conclusions

In the past 5 years of epidemiological analysis of leptospirosis in Costa Rica, we can conclude that 1 of every 10 samples sent to INCIENSA was positive for the spirochete. This data is important because despite the low number of reported positive cases, there were multiple suspect cases. This high clinical suspicion is similar to other febrile diseases such as dengue, chikungunya, and Zika which mimic leptospirosis in disease presentation [15].

**Fig. 2:**
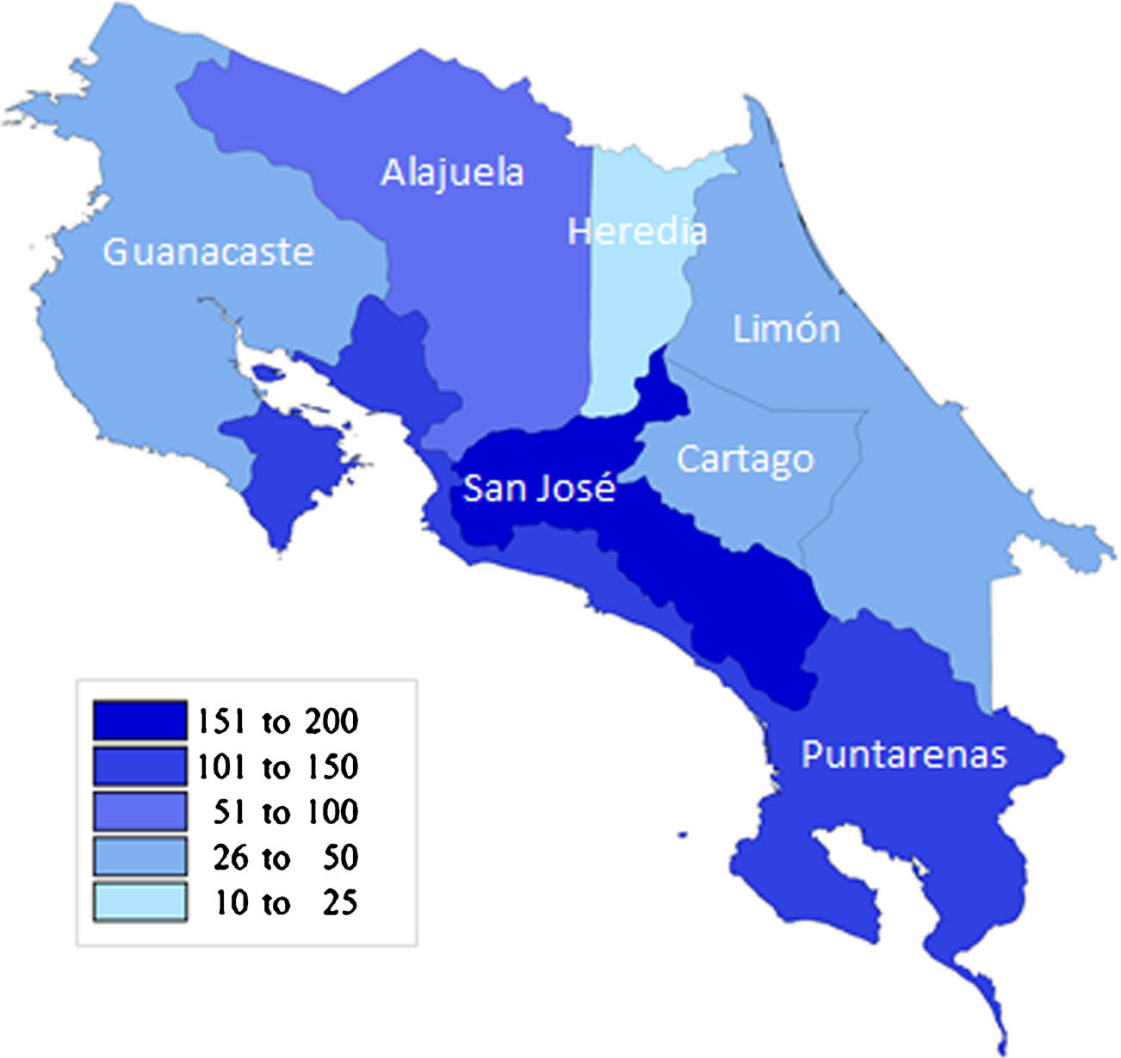
Positive cases by province, Costa Rica, 2011–2015. Source: Self-evaluated using INCIENSA data

The differences of positive cases of leptospirosis according to season suggest a strong environmental influence with the leptospirosis spirochete exposure in rainfall, flooding, and also in household environments. Upon reviewing Fig. 1, we see that leptospirosis cases surge in October–December of 2011, which aligns with severe flooding that occurred in the country in the end of October. The regions most afflicted by the flooding and landslides were San Jose, Alajuela, and the Pacific Coast, also consistent with Fig. 2’s map of cases [16]. Upon severe flooding during October of 2015, however, cases of leptospirosis were few. In fact, two other seasons reported higher cases of leptospirosis than during the October 2015 floods [17]. The rainy season in Costa Rica is typically May through November, yet in January through March of 2012, 2013, and 2014, more cases were seen during that “dry” season than any other season of those years. This realization points to several conclusions: First, flooding and rainfall certainly may affect leptospirosis cases as seen in the 2011 flood data. Second, flooding and rainfall are not always indicators of high leptospirosis cases, suggesting other environmental factors play a role in transmission. Leptospirosis has been found present in puddles, containers, animal troughs, rivers, canals, and even drinking water [18, 19]. It has also been found to be linked with people with exposure to rodents, domestic animals, and dogs [18, 19]. These findings point toward other environmental influences as playing an important and often overlooked role in leptospirosis.

In addition to environmental influences, the evidence is quite strong in pointing toward several risk factors for leptospirosis: being a male in productive, working years. This evidence was so strong that several age groups saw upwards of a tenfold difference between male and female confirmed cases. In males, the ages with the greater number of positive cases are those between the ages of 21 to 30 years old. The number of positive cases starts to increase, however, after age 11 and decrease gradually after age 50. Females are similar to males with respect to age and gender: after age 11, positive samples begin increasing, peaking between the ages of 51 and 60 years old, and abruptly decreasing afterward.

Overall, our data supports a growing body of evidence that leptospirosis is an endemic disease in Costa Rica. Clinicians should be aware that epidemiologic risk factors vary by region, with working age men being most likely to present with clinical disease. While flooding was likely an important cause of outbreaks, our epidemiological data of dry seasons also demonstrated consistent case burdens. Future studies should investigate the environmental and occupational influences on disease transmission in an effort to decrease disease burden in Costa Rica.

## Abbreviations

EBAIS: Government-sponsored community health clinics (Equipo Básico de Atención Integral en Salud)
INCIENSA: Costa Rican Ministry of Health (Instituto Costarricense de Investigación y Enseñanza de Nutrición y Salud)
CCSS: Costa Rican Department of Social Security (Caja Costarricense del Seguro Social)
MAT: Microscropic agglutination test
PCR: Polymerase chain reaction
